# Insomnia and the role of postmigration stress among Syrian refugees

**DOI:** 10.1192/j.eurpsy.2022.1625

**Published:** 2022-09-01

**Authors:** M. Straiton, A. Nissen

**Affiliations:** 1 Norwegian Institute of Public Health, Department Of Mental Health And Suicide, Oslo, Norway; 2 Norwegian Centre for Violence and Traumatic Stress Studies, N/a, Nydalen, Norway

**Keywords:** Refugees, Insomnia, Postmigration stress, Forced migration

## Abstract

**Introduction:**

Research on the prevalence of and risk factors for insomnia among refugee populations is limited and tends to focus on pre-migratory trauma. Yet, post migratory stressors are just as important for mental health and may also relate to insomnia.

**Objectives:**

Objective: To determine the association between different post-migration stressors and insomnia among Syrian refugees living in Norway.

**Methods:**

We used data from the REUFGE study, a cross-sectional survey with 902 Syrian refugees who arrived in Norway between 2015 and 2017. Insomnia was measured with the Bergen Insomnia Scale and post-migrant stress with the Refugee Post-Migration Stress Scale (RPMS). We applied logistic regression analyses to investigate the association between seven different postmigration stressors and insomnia after controlling for demographics, traumatic experiences and post traumatic stress symptoms.

**Results:**

Of the 873 participants who completed questions on insomnia, 515 (41%) reported insomnia. There was no significant difference between men and women. The most commonly reported postmigration stressors were *Competency Strain* [SML1]*, Family and Home Concerns*, and *Loss of Home Country.* After controlling for demographics, traumatic experiences and post-traumatic stress symptoms, *Financial Strain*, *Loss of Home Country, Family and Home Concerns* and *Social Strain* were still associated with higher odds of insomnia.

**Conclusions:**

Resettlement difficulties are related to poorer sleep among refugees. Measures to improve the social conditions and financial concerns of refugees in receiving countries could potentially reduce insomnia among refugees which in turn, may benefit mental and physical health.

**Disclosure:**

No significant relationships.

